# Deep Venous Reconstruction: A Case Series

**DOI:** 10.7759/cureus.1518

**Published:** 2017-07-26

**Authors:** Sebastian Kosasih, Hayley Moore, Tristan R Lane, Alun H Davies

**Affiliations:** 1 Academic Section of Vascular Surgery, Imperial College School of Medicine

**Keywords:** deep venous reconstruction, deep venous bypass, palma, femoral-femoral crossover bypass, may-husni, saphenopopliteal bypass, chronic venous disease, chronic venous insufficiency

## Abstract

Objectives

This study aims to review a case series of deep venous reconstruction procedures performed at one centre by a single consultant.

Methods

A retrospective review of deep venous reconstruction procedures performed by a single consultant from 1994 to 2013 was carried out and all notes were reviewed for outcomes. A 58-month cumulative patency rate was calculated using Kaplan-Meier survival analysis.

Results

Nineteen patients underwent deep venous reconstruction procedures including the Palma bypass, May-Husni bypass, femoral vein transposition and axillary vein transplant techniques from 1994 to 2013. Eleven patients were male and eight were female with a mean average age of 45.2 years (range 29-63). Clinical severity of disease ranged from C3 to C6, and 16 patients had a confirmed history of deep vein thrombosis. Cumulative primary patency rate for all reconstructions at 58 months was 89.5%, with two patients occluding and 17 remaining patent at last follow-up.

Conclusion

Deep venous reconstructions, particularly the Palma and May-Husni procedures, are feasible and can have good outcomes in patients failed by endovascular techniques and other more conservative therapies.

## Introduction

Chronic venous disease (CVD) is a spectrum of clinical presentations including varicose veins and chronic venous insufficiency (CVI) [1]. CVD is the result of abnormally functioning superficial or deep veins. Classified as primary or secondary, the former indicates that no trigger mechanism of venous dysfunction can be identified. This suggests that intrinsic biochemical and structural abnormalities of the vein walls are to blame, and is confined purely to reflux. The latter points to a triggering event as the cause of dysfunction and is most commonly due to deep vein thromboses (DVTs) causing damage to vein walls and valves, the clinical manifestation of which is known as post-thrombotic syndrome (PTS) [2]. It is often a combination of both refluxing and obstructive disease. CVD is common with a prevalence of approximately 83.6% worldwide, albeit with a predisposition towards milder disease stages [3]. There are several efficacious treatments available for superficial venous disease or varicose veins [4], whereas treatment of deep CVI, particularly by surgical means, is more open to debate [5].

Over one billion dollars per year is spent in the United States on CVI and venous ulcer treatment [1]. The most common treatments for deep CVI are conservative, such as compression stockings or elevation of the affected leg in order to improve venous return [6], alongside optimal management of systemic conditions which affect ulcer healing such as diabetes mellitus. Additionally, weight loss, exercise and physical therapy to improve the mobility of the ankle joint have been shown to help [2, 7]. Pharmacological treatments are also available, including venoactive drugs which act on venous tone to reduce venous hypertension by inhibiting changes in the microcirculation [8].

The current gold standard of management, particularly in cases of obstructive lesions is endovascular stenting, a minimally invasive surgery performed percutaneously to introduce a stent into a narrowed or thrombosed vein [2]. Unfortunately, stenosis and thrombosis of stents may still occur, and in these cases more invasive surgery may need to be considered.

Such surgery can be carried out on either the superficial or deep veins. Superficial vein surgery may be beneficial, although some studies [9-10] dispute this. However, previous work has been limited by trial intervention non-compliance. Hence, superficial vein surgery is often attempted for ulcer healing, even though there is limited evidence available.

On the other hand, evidence for deep venous surgery is lacking. There are many surgical options available and outcomes are variable. All deep vein reconstructions have the potential to lead to excellent outcomes, and offer an additional treatment in those for whom the more conservative treatments have failed. However, these technically challenging, highly invasive procedures are only performed in a few specialist centres by experienced surgeons. Thus, there is a paucity of literature relating to results with existing case series having variable outcomes.

This study aims to present a case series of deep venous reconstructions performed by one surgeon at a single centre, in order to add to the current knowledge base and inform clinicians of the potential outcomes and efficacy of this niche area of surgery.

## Materials and methods

We performed a retrospective review of 19 patients who underwent deep venous reconstruction for either refluxing or obstructive venous disease between 1st January 1994 and 1st March 2013. Data was collected using local electronic operation records from 4th April 2008 to 19th February 2013, patient correspondence (2006 to present), the private patient database, operating theatre books (1994 up to but not including 2009), and clinical notes. Patients on whom any deep venous reconstruction from the iliac veins distally had been performed, for any pathology, were included. Theatre books were searched manually, and electronic operation records, patient correspondence and the private patient database were searched using Windows search function for the terms: ‘recon’; ‘valvuloplasty’; ‘external valve banding’; ‘transplant’; ‘transposition’; ‘translocation’; ‘neovalve’; ‘maleti’; ‘plagnol’; ‘saphenopopliteal’; ‘bypass’; ‘May-Husni’; ‘palma’.

Exclusion criteria included deep venous reconstructions involving the inferior vena cava, arterial procedures, venous reconstruction of the arm, unperformed deep venous reconstructive procedures, ‘self-palmas’ (collateralisation in a similar pattern to the Palma procedure), palmar-plantar erythrodysesthesia, and renal or other organ transplants.

Notes for included patients were requested for follow-up, with venous duplex scan at last follow-up being used to assess patency where possible, or the last known status as per correspondence if not. The end point was either patent or occluded. A combination of patient notes and correspondence were used to establish pre-operative CEAP scores. Using only the ‘C’ or clinical classification of CEAP, ranging from 1 to 6, patients were classified according to disease severity. Sex, indication for surgery, risk factors, previous DVTs, ulcers and anticoagulation regimens were also recorded.

**Figure 1:**
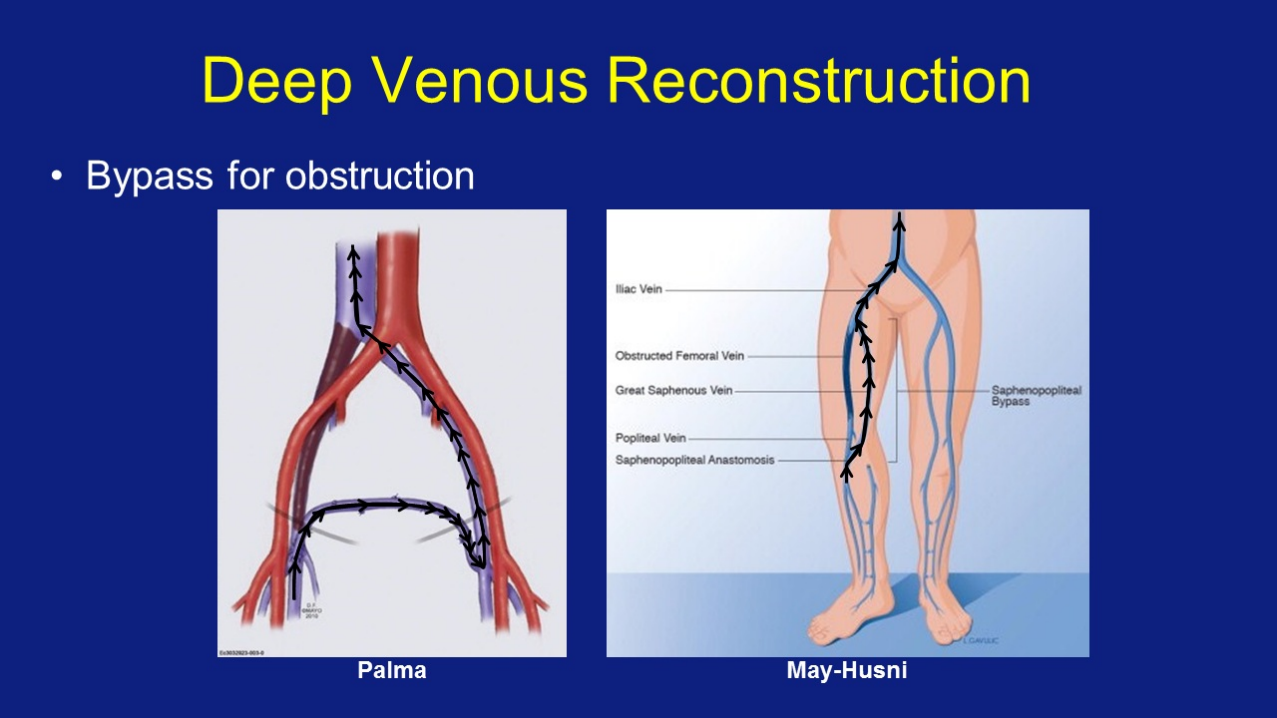
The Palma (femoro-femoral bypass) and May-Husni (saphenopopliteal bypass) procedures.

The primary outcome measure of this case series was the 58-month cumulative primary patency rate. Subgroup analysis of Palma and May-Husni procedures (Figure 1) was also performed, and CEAP score reductions and correlation between CEAP score at presentation and patency length, if still patent at last follow-up, were examined. The 1st of May 2013 was used as the end point.

Statistical analysis

IBM SPSS Statistics 21 (Armonk, NY) for Windows was used for Kaplan-Meier analysis to calculate cumulative primary patency rates as well as the number at risk at 12-month intervals. It was also used for the two-tailed Spearman’s rank order correlation and Mantel-Cox log rank tests. The significance level for Spearman’s and Mantel-Cox analyses was set at p < 0.05.

## Results

**Figure 2:**
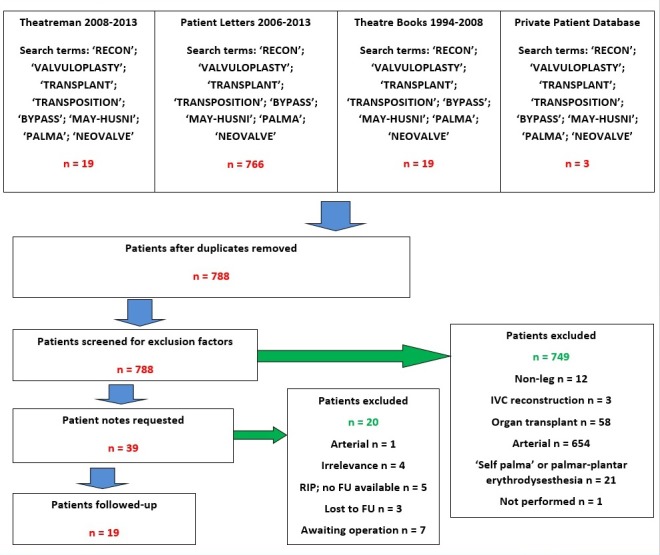
Flow chart of case retrieval.

Nineteen patients (11 male, eight female), mean age 45.2 years (range 29-63) underwent 28 deep vein reconstructive procedures for either refluxing or obstructive deep venous disease (Figure 2). Sixteen patients had confirmed previous DVTs, and one case was caused by femoral occlusion secondary to trauma and scarring. In the final two previous DVT could not be confirmed.

Twenty procedures were performed on male patients and eight on females. Of these patients, three males had had multiple procedures before – one had undergone a previous Palma procedure (femoro-femoral bypass), while another had had an axillary vein transplant to the right superficial femoral vein (SFV), May-Husni (saphenopopliteal bypass), and an earlier right leg deep venous bypass. The third had had five previous procedures: one Palma with subsequent revision, a deep femoral venoplasty with polytetrafluoroethylene (PTFE) graft, and two May-Husni procedures. Seventeen patients had complete follow-up data with duplex scans. Patency for those whose scans were not available was determined clinically at most recent follow-up. Risk factors for deep CVI found in the 17 patients with complete follow-up information included four with a history of smoking, two who were overweight, three with a history of limb trauma, one with a history of pulmonary embolism, and three with thrombophilias. One patient had idiopathic thrombocytopenic purpura. All procedures were elective, and all patients were anticoagulated on discharge. IV heparin was used peri-operatively, and the anticoagulant given on discharge was warfarin, aside from one patient who was given dabigatran and aspirin due to warfarin allergy.

CEAP scores at presentation were available for 17 patients, ranging from C3 to C6. Four cases had leg swelling and venous claudication (C3) secondary to CVI, and two had skin changes (C4). A further seven presented with active venous ulceration (C6), and four had healed ulcers (C5).

Pre-operative duplex scans were available for 12 patients, all of whom had secondary aeitiology with 11 cases of PTS and one case of trauma scarring. This included two patients with partially occluded obstructive lesions, three with completely occluded obstructive lesions, three cases with reflux (one axial reflux, a femoral segmental and a long saphenous segmental reflux) and four with a mixed picture. From the pre-operative scans, two iliac veins were occluded to some degree and one was incompetent. In the femoral veins, there were three incompetent segments and nine occluded ones. There were a total of four problems in the popliteal veins, with three incompetencies and one occlusion. Additional segments of reflux were: two long saphenous, one short saphenous, and three posterior tibial. Six patients had multilevel disease. Post-operative duplex scans were available for 16 patients.

Of the procedures performed, there were five Palmas (one with PTFE graft), four May-Husnis, three inline femoroiliac bypasses (one with PTFE graft), one inline bypass from superficial to common femoral vein, one endophlebectomy on the external iliac vein, one femoral internal valvuloplasty, two axillary vein transplants (one with PTFE wrap), and two femoral vein transpositions. At least 11 of the 19 patients are known to have used compression therapy previously, but this failed to improve symptoms.

Three patients had previously failed stenting (two iliac and one inferior vena cava), four had undergone superficial venous surgery for varicose veins or other symptoms, and four had previous deep venous reconstructions.

Early results at 30 days

Patients were anticoagulated and had duplex scans post-operatively to ensure graft patency. In the post-operative period, there were no clinical signs of pulmonary embolisms, and no other complications noted. There was no post-operative mortality recorded in 30 days. One patient occluded instantly and preferred not to undergo a revision procedure, and all other procedures remained clinically patent in this period. Therefore, 30-day primary patency was 94.7%.

Late results

**Figure 3:**
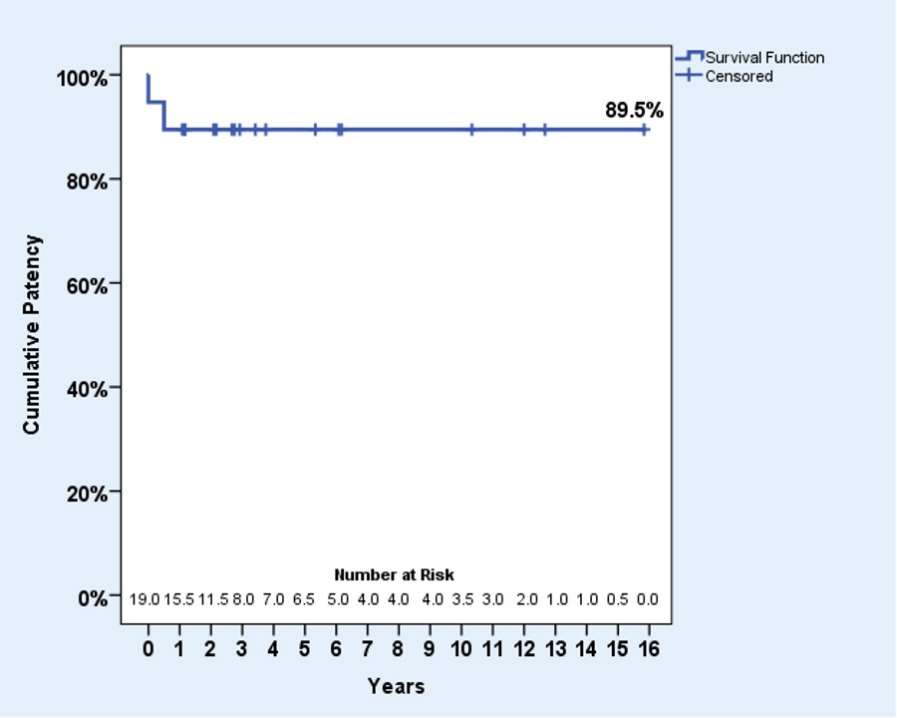
Kaplan-Meier survival plot for 58-month cumulative primary patency.

Overall median follow-up time was 35 months (interquartile range (IQR) 54 months) and mean follow-up time was 58 months. When comparing between procedures, individuals’ follow-up times or time until occlusion (months) ranged from 0 to 144 for Palma, 13 to 190 for May-Husni, 6 to 74 for inline bypass, 33 to 152 for axillary vein transplant, 35 to 124 for femoral vein transposition, 25 for endophlebectomy, and 41 for internal valvuloplasty. Thus, the primary outcome measure used was a 58-month cumulative primary patency rate. At 58 months, there had been two occlusions and 17 patent grafts at last follow-up (censored). This gave a primary patency from Kaplan-Meier analysis of 89.5% (CI: 68.6-97.1%) as in Figure 3. It should be noted that due to censoring (that is, patients who were patent at last follow-up and have not re-presented to our team since) there is a possibility that some occlusions occurred but were not recorded or clinically recognised.

**Figure 4:**
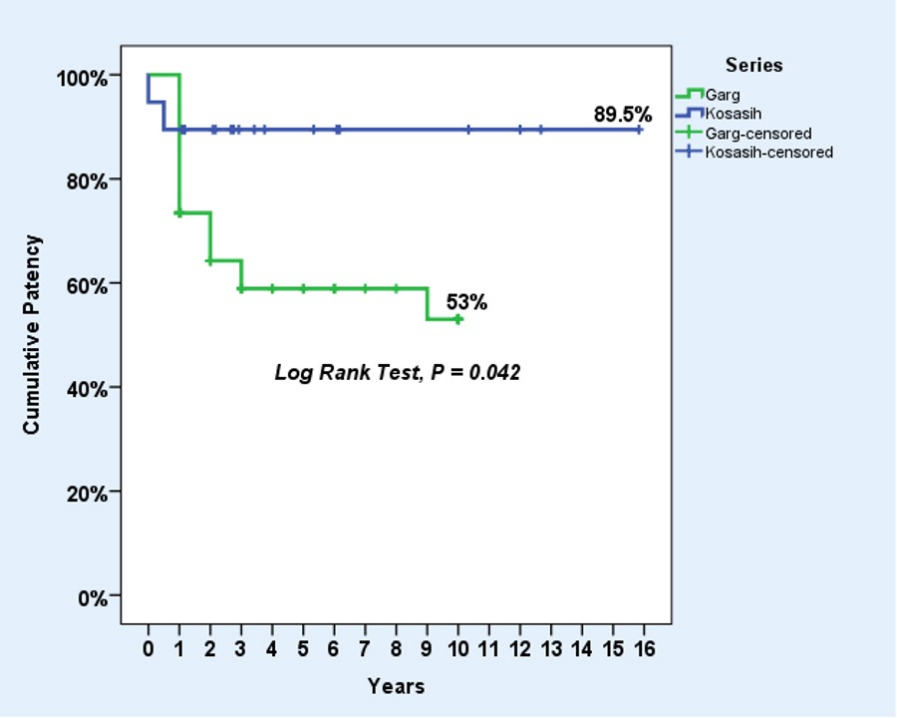
A comparison of results with the Mayo Clinic.

This was compared to a similar collection of reconstructions by Garg, et al. (Mayo Clinic) [11], and a log rank test was done, giving χ^2^ = 4.15 and P = 0.042, showing a significant difference between the two series (Figure 4).

Three procedures were performed with PTFE graft (a palma, femoroiliac bypass and axillary vein transplant wrap), and of these the Palma procedure occluded, giving a patency rate of 66.7% (CI: 20.8-93.9%).

CEAP scores at presentation were available for 17 patients (89.5%) and at follow-up for seven of the 19 patients (36.8%). Of these latter seven, six had improved from presentation, including one patient who presented with an ulcer which had subsequently healed. Interestingly, one of the improved CEAP scores was found in a patient with an occluded graft, while in the one case in which there was an unimproved CEAP score, the patient had a patent graft. In those for whom we had data, clinical benefit was found in 85.7%, with a median CEAP reduction of 1 (IQR 2) in these seven patients. With regards to the whole group, CEAP scores at presentation for the 17 available had a median of 5 (IQR 2), whereas CEAP scores available at follow-up had a median of 3 (IQR 4), giving an overall reduction in median CEAP score of 2. Additionally, a two-tailed Spearman’s rank order correlation was performed in order to find a correlation between CEAP scores at presentation and patency length. However, no correlation was found, with an rs = -0.177, (p = 0.498).

Subgroup analysis

Five patients who underwent Palma procedures, four who had undergone May-Husni procedures and four with inline bypasses were compared using Kaplan-Meier survival analysis for 58-month cumulative primary patency rates.

The 58-month primary patency of the Palma procedure was 80.0% (CI: 37.6-96.4%) with one instant occlusion of the Palma with PTFE graft. The May-Husni had a primary patency rate at 58 months of 100% (CI: 51.0-100%), while one femoroiliac inline bypass occluded to give a 75.0% (CI: 30.1-95.4%) primary patency. Overall, the combined 58-month primary patency of the Palma, May-Husni and inline bypass reconstructions was 84.6%.

A list of other procedures and outcomes is as follows: two axillary vein transplants; two femoral vein transpositions; one femoral internal valvuloplasty; one iliac endophlebectomy, all of which remained patent and competent. A breakdown of the different procedures may be found in Table 1.

**Table 1:** Breakdown of procedures in series by procedure type.

Procedure type	Number of procedures in series	Primary patency rate (%)	Median follow-up in months	Mean age of patient (Years)
Palma bypass	5	80	32	44
May-Husni bypass	4	100	29.5	42.5
Inline bypass	4	75	45	48.25
Axillary vein transplant	2	100	92.5	50
Femoral vein transposition	2	100	79.5	38.5
Endophlebectomy	1	100	25	36
Internal valvuloplasty	1	100	41	63

## Discussion

In this case series, we have demonstrated that good outcomes can be achieved from deep venous reconstructions when performed by an experienced surgeon.

However, open reconstructive procedures for CVI are not commonly performed due to their invasive nature and the experience required of the surgeon. Consequently, there is a paucity of literature on outcomes. Evidence available suggests that primary disease obtains better outcomes than PTS when treated by deep venous surgery [5, 12], though this is disputed [13].

With primary deep reflux, internal and external valvuloplasties are options. The internal method results in approximately 70% of ulcers healing [12, 14-16] versus 50% for external [12].

A common procedure used for secondary post thrombotic reflux is axillary vein transplant [17]. Ulcer healing rates for these procedures are varied with 43%->60% 10-year ulcer-free survival (UFS) [12, 15-17]. Transposition and neovalve procedures are less commonly performed. Results for venous (femoral) transpositions range from 44% to 75% UFS [15-16, 18-19].

Neovalve studies are promising – Maleti’s technique has been shown to give 89% ulcer healing with 95% valve competency [20] while Plagnol’s technique had 90% patency [21], but short follow-up is a limitation of these studies.

For obstructive disease, the Palma procedure is considered the best option in those who fail iliac vein stenting [22]. Results in the literature are summarised in Table 2 [11,23-26], and appear to be comparable to our results.

**Table 2:** Summary of Palma procedure results from the literature (current series in bold).

Author	Follow-up (months)	Patients	Patency
Husni (1978)	7-144	78	73%
Hutschenreiter (1979)	6-78	20	44%
Aburahma (1991)	66 (mean)	24	75%
Jost (2001)	10-62	18	83%
Garg (2011)	0.5-252	25	78%
Kosasih (2017)	0.5-144	5	80%

**Figure 5:**
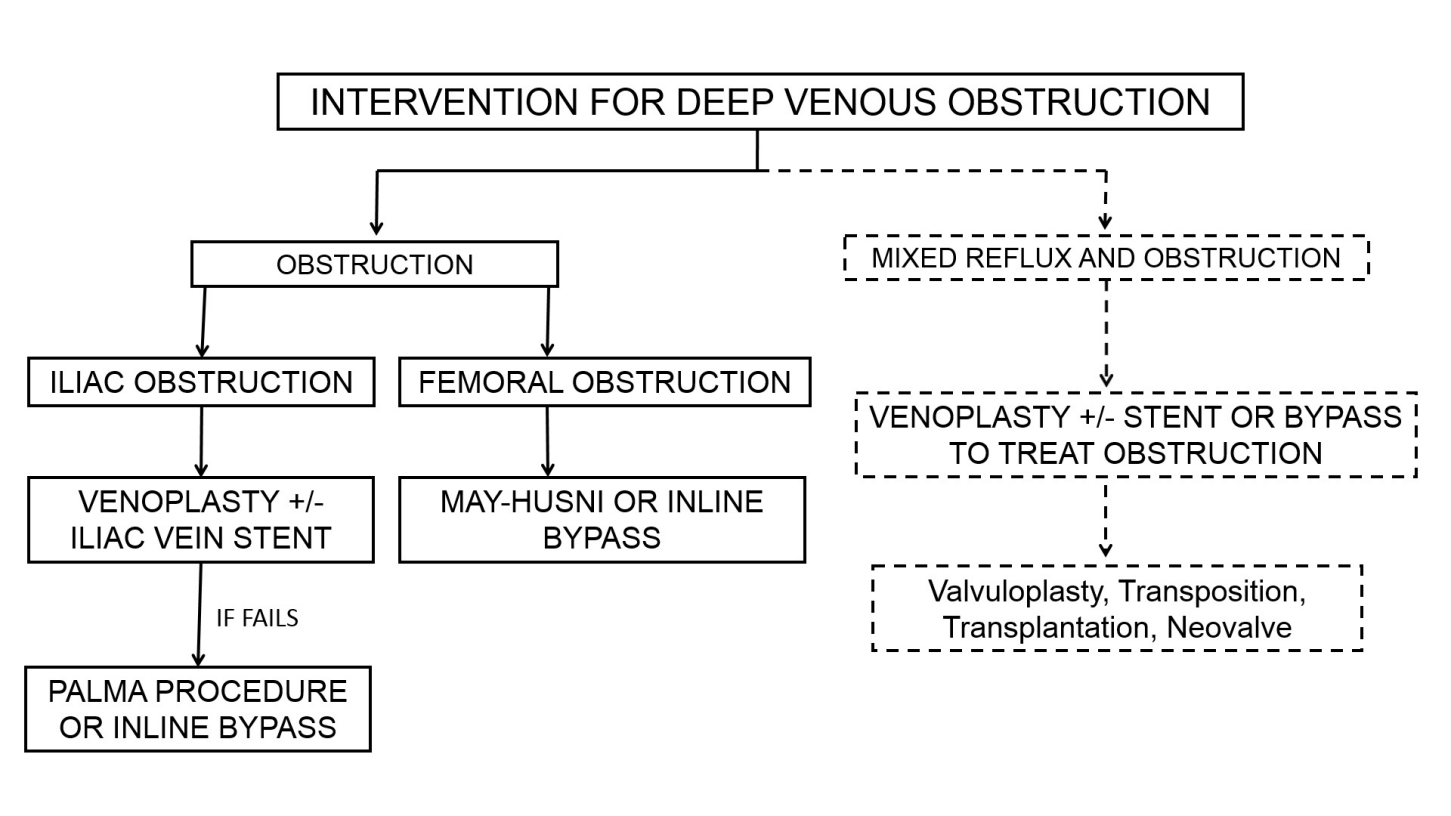
A flow chart demonstrating the current management of deep venous obstruction.

The May-Husni saphenopopliteal bypass is indicated for patients with femoral vein obstructions, with previous results being less successful than the current series [23-24,27]. A summary diagram of the suggested management for deep venous reconstruction is shown in Figure 5.

Limitations of the literature include short, inconsistent follow-up times. Though as with other retrospective studies this is inevitable, objective comparison of treatments and long-term benefits are consequently difficult. Equally, many studies are not recent: historical outcomes may not necessarily hold true anymore, especially given the advances in surgical technology which have occurred in recent years.

In this case series, the majority of included patients had C4-6 disease, and since milder cases of CVD may be adequately controlled with compression stockings [6], this study can only be applied to these patients.

The key finding of this series was a 58-month cumulative primary patency of 89.5%, which is comparable to Garg’s Mayo Clinic patency of 53.0%, recognising that the latter has a much larger number of patients [11]. The log rank test showed a significant difference between these series with p = 0.042 (Figure 4), suggesting that comparable results are achievable even if procedures are performed less frequently. However, it should be noted that the combination and types of reconstructions in the Mayo Clinic series, though similar, were not exactly the same as in this series, so the comparison is indirect.

Primary patencies of the Palma, May-Husni and inline bypass techniques were also investigated. The 58-month primary patency of the Palma was 80% and the May-Husni’s was 100%, while the inline bypasses achieved 75%. These results are comparable to those found in the literature [18, 26-27]. However, in all groups numbers are small – five patients in the Palma category, and four in the May-Husni as well as the inline bypass, a significant limitation of the study.

While there was some expectation that higher CEAP scores may have been correlated with shorter patency length, no correlation was found between CEAP scores at presentation and patency length, suggesting deep venous reconstruction should not be used in an algorithmic fashion according to CEAP scores. Rather, it should be tailored to each patient’s needs as outcomes are variable. A limitation of this analysis was that CEAP scores on presentation were unavailable for some patients.

Synthetic PTFE grafts may have worse outcomes due to thrombogenicity and poor haemodynamic properties [28]. Two PTFE grafts were used in this series, and one (a Palma) of these occluded instantly, though with only two cases we cannot really draw any conclusions. Jost’s series [26] had 17 PTFE grafts put in for various procedures, including three cross-femoral Palma bypasses. Their overall PTFE two-year patency was 45%, but all three Palmas with PTFE grafts occluded. Hence, although PTFE grafts are only used in Palmas for those without suitable vein grafts, PTFE grafts should only be used in long bypasses such as the Palma as a second line as the combination of poor haemodynamics, a long conduit, and thrombogenicity is an insurmountable issue. Short bypass outcomes in other series are more encouraging [11].

Both occlusions in this series occurred early on, with one occluding instantly and the other at six months. The rest remained patent at last follow-up, indicating a pattern in which patients tend to either occlude early or remain patent for a long time. This could reflect variations in patient anatomy, indicating some patients may not be suitable for these procedures due to factors such as vein calibre. In order to fully evaluate this, there is a need for ongoing critical analysis of long term results of bypasses in PTS.

Ideally, a correlation of patency and clinical outcome would have been performed. Unfortunately, information on clinical outcome was lacking. However, of the data available, one patient who remained patent did not have any symptomatic reduction (swelling persisted). Conversely, another patient who occluded noticed a clinical improvement (a healed ulcer). The remaining five for whom we have clinical outcomes were all patent and experienced symptomatic reduction.

Limitations of this study included the fact that these procedures are rare, the timescale is long and the data, therefore, spans different eras of recording, producing difficulty in finding clinical information. Due to the complicated nature of the surgery and the experience required to perform the procedures, there are small numbers throughout, therefore comparison of individual techniques is difficult. Additionally and perhaps most importantly, there were those limitations associated with the retrospective nature: the small number of cases, variable patient selection and its influence on outcomes, variable follow-up and incomplete clinical outcome data which made meaningful statistical analysis difficult. Furthermore, the high number of censored cases which may have occluded after last follow-up, incomplete data from old records, and a heterogeneous mixture of procedures exacerbated this. Imaging data from pre-operative duplex and ultrasonography studies were not available for the majority of patients, resulting in a more limited understanding of the extent of disease. Similarly, a lack of data on previous compression therapy use in some patients was a problem. Nevertheless, this case series has value in contributing to the current literature on the outcomes of deep venous surgery, ensuring that it remains a part of the armoury for treating patients with deep venous disease who have been failed by other methods.

Future studies could look into a correlation between patency and clinical benefit, or look for anatomical (or other) factors which affect and correlate with outcomes. This should be done with a view to understanding which patients could benefit from these invasive procedures.

## Conclusions

This study confirms that deep venous reconstruction is feasible, and the Palma and May-Husni procedures especially are alternatives which can have good outcomes in terms of both patency and clinical improvement, for patients with femoroiliac obstructive lesions failed by endovascular techniques including venous stenting, or conservative methods.
